# The Vast and Varied Global Burden of Norovirus: Prospects for Prevention and Control

**DOI:** 10.1371/journal.pmed.1001999

**Published:** 2016-04-26

**Authors:** Benjamin A. Lopman, Duncan Steele, Carl D. Kirkwood, Umesh D. Parashar

**Affiliations:** 1 Division of Viral Diseases, National Center for Immunization and Respiratory Diseases, Centers for Disease Control and Prevention, Atlanta, Georgia, United States of America; 2 Enteric & Diarrheal Diseases, Bill & Melinda Gates Foundation, Seattle, Washington, United States of America

## Abstract

Globally, norovirus is associated with approximately one-fifth of all diarrhea cases, with similar prevalence in both children and adults, and is estimated to cause over 200,000 deaths annually in developing countries. Norovirus is an important pathogen in a number of high-priority domains: it is the most common cause of diarrheal episodes globally, the principal cause of foodborne disease outbreaks in the United States, a key health care–acquired infection, a common cause of travel-associated diarrhea, and a bane for deployed military troops. Partly as a result of this ubiquity and burden across a range of different populations, identifying target groups and strategies for intervention has been challenging. And, on top of the breadth of this public health problem, there remain important gaps in scientific knowledge regarding norovirus, especially with respect to disease in low-income settings.

Many pathogens can cause acute gastroenteritis. Historically, rotavirus was the most common cause of severe disease in young children globally. Now, vaccines are available for rotavirus and are universally recommended by the World Health Organization. In countries with effective rotavirus vaccination programs, disease due to that pathogen has decreased markedly, but norovirus persists and is now the most common cause of pediatric gastroenteritis requiring medical attention. However, the data supporting the precise role of norovirus in low- and middle-income settings are sparse. With vaccines in the pipeline, addressing these and other important knowledge gaps is increasingly pressing.

We assembled an expert group to assess the evidence for the global burden of norovirus and to consider the prospects for norovirus vaccine development. The group assessed the evidence in the areas of burden of disease, epidemiology, diagnostics, disease attribution, acquired immunity, and innate susceptibility, and the group considered how to bring norovirus vaccines from their current state of development to a viable product that will benefit global health.

## Abstract 2

Lopman and colleagues outline key findings from the PLOS Norovirus Collection. The global burden falls hardest on children, and efforts to develop vaccines focus primarily on this vulnerable group.

**Summary Points**

- Diagnostic improvements have fundamentally changed our understanding of norovirus. The current evidence suggests that disease burden of norovirus is high, second only to rotavirus as a cause of severe acute gastroenteritis in children in developed countries, and that it is a key cause of diarrhea-associated morbidity and mortality worldwide.
- Young children experience the highest incidence of disease; severe outcomes are most common among young children and the elderly.
- Immunity is of limited duration and is strain- or genotype-specific, with little or no protection conferred across genogroups.
- Innate susceptibility to noroviruses is determined by the host’s genetics of glycan expression; individuals with a functional FUT2 gene (known as secretors) have greater susceptibility to certain common viruses.
- Recent progress has been made in the development of in vitro cell culture for norovirus as well as in the identification of candidate immune correlates of protection.
- Norovirus vaccines are steadily moving through the development pipeline. All of these products are based on the production of virus like particles (VLPs) or P particle subunit in expression systems. Initial human challenge studies have demonstrated safety, immunogenicity, and efficacy.
- One of the challenges for developing targeted interventions, including a norovirus vaccine, is that many distinct population groups, based on demographics (e.g., children, elderly) or risk (e.g., food handlers, military, travelers, health care workers), are affected.

## Introduction

Significant progress has been made towards the control of diarrheal diseases. Global diarrheal deaths for all ages have declined dramatically, from an estimated 2.6 million annually in 1990 to approximately 1.3 million in 2013 [1]. Over the same period, improvements specifically for children under 5 years of age have been most impressive, with rates of decline in diarrheal deaths at around 5% per year, in absolute numbers, to an estimate of 578,000 deaths in 2013 [2]. Diarrheal disease is now the fourth most common cause of mortality overall, but morbidity has not changed at the same pace, with diarrheal disease remaining the second most common cause of morbidity worldwide in children under the age of 5 years [3]. Many bacterial and parasitic pathogens are controllable through improvements in water, sanitation, and hygiene, facilitated by economic development, as is evidenced by the rapid declines in mortality; rotavirus disease has been significantly impacted through vaccination in virtually every setting where it has been introduced, and can be further impacted with additional introduction of rotavirus vaccines in countries [4].

Norovirus is ubiquitous, associated with 18% (95% CI: 17%–20%) of diarrheal disease worldwide, with significant burden of disease in high-, middle-, and low-income settings. It is estimated to cause 212,000 deaths annually worldwide; approximately 99% of these are estimated to occur in middle- and high-mortality countries [5]. According to these estimates, norovirus is the most common cause of diarrheal cases across for all ages, the second most common cause of diarrheal death in children under the age of 5 years, and the most common cause of diarrheal death over 5 years of age, with similar patterns across WHO regions. So while the overall mortality risks are likely to be much lower in high-income settings, high incidence of disease appears universal. For example, the Malnutrition and Enteric Disease Study (MAL-ED), conducted in eight low- and middle-income countries, found norovirus to be the first and second most common cause of diarrheal disease in the first and second year of life, respectively [6]. And in high- and middle-income countries with successful rotavirus vaccination programs, norovirus is the most common cause of pediatric gastroenteritis [7,8].

The increase in norovirus-related research over the last 15 years has been tremendous, concurrent with improvements in and more widespread availability of diagnostic methods. Included in this body of work are fundamental advances in our understanding, ranging from better burden-of-disease estimates [5,9,10], to the first demonstration of in vitro cell culture [11], to elucidation of key interactions that noroviruses have with other agents in the microbiome [11–13], to the demonstration that vaccination against human norovirus infection and disease is possible [14,15]. While many challenges remain before norovirus can be considered “controllable,” the confluence of recent advances gives us optimism. To this end, in February 2015, Centers for Disease Control and Prevention (CDC) and the Bill & Melinda Gates Foundation (BMGF), led by a scientific organizing committee from the areas of government, academia, and philanthropy, convened a symposium in Atlanta, Georgia, United States, with representatives from all these sectors in addition to vaccine developers. Our charge was to review the most up-to-date knowledge on norovirus with the aim of identifying key gaps and detailing the most critical studies to address them, all with the ultimate goal of guiding the development of a norovirus vaccine for the populations that stand to benefit most: children in the developing world. The full report from the convening can be found at http://www.cdc.gov/norovirus/downloads/global-burden-report.pdf. Many of the papers in this PLOS Collection were either presented at or inspired by the meeting.

## Critical Knowledge Gaps

1. Basic epidemiological and disease burden data are lacking, especially from developing countries. There remains uncertainty and some scientific controversy in defining the norovirus disease burden.
2. With sensitive real-time quantitative PCR (RT-qPCR) assays, norovirus is frequently detected in stool of healthy individuals, complicating the interpretation of diagnostic results and disease attribution.
3. Understanding of natural immunity to norovirus remains incomplete.
4. The implications of genetic susceptibility for population health, viral evolution, and vaccines remains unclear.
5. Genotype-specific immune responses and antigenic variation of norovirus suggest that a polyvalent vaccine will be needed and may require updating when new pandemic strains emerge.
6. There is limited understanding of the relative roles that different age groups play in virus transmission. Better data coupled with appropriate models could help to devise vaccination strategies that lead to the greatest benefits at the population level.

## Insights from this Collection

### Global Economic Burden

In a recent PLOS Collection, the World Health Organization’s Global Estimates of the Burden of Foodborne Disease in 2010 were published. These estimates position norovirus as the most common cause of cases of and deaths from foodborne diarrhea disease and the fourth greatest burden in terms of disability-adjusted life years (DALYs). Norovirus was estimated to cause 684 million (95% Uncertainty Interval 491–1,112 million) episodes of diarrheal disease and 212,000 deaths, annually, for all ages and from all modes of transmission [5,10].

In our current Collection, Bartsch et al. extend those findings to consider global economic impacts of norovirus [16]. They estimate that globally, norovirus results in an economic burden of US$4.2 billion (95% UI: US$3.2–US$5.7 billion) in direct health system costs and US$60.3 billion (95% UI: US$44.4–US$83.4 billion) in societal costs annually. Two-thirds of that burden is a result of disease in children under the age of five years. Low-, middle-, and high-income countries all have a considerable economic burden, indicating that norovirus gastroenteritis is a truly global economic problem.

### Local Burden Data

While there is clearly a lack of local, national, and regional studies from developing countries on the epidemiology and molecular diversity of norovirus, those gaps are beginning to be filled. In their systematic review, Mans et al. included data on 19 studies from 14 African countries. Overall, in these studies, norovirus was associated with 13.5% of diarrheal disease in children, and GII.4 strains predominated in the majority of studies. The authors identified lack of data in older children and adults as a critical gap in Africa [17]; the same gap exists for low- and middle-income settings globally [9].

A number of papers in this collection also offer new epidemiological data from specific populations from Asia, Africa, and North and South America. Shioda et al. present some of the first age-specific community and outpatient incidence rates of diarrheal disease associated with norovirus (as well as sapovirus and astrovirus) in Kenya [18]. Their community-based incidence estimate is about twice that of estimates from the US, United Kingdom, and the Netherlands, suggesting higher overall incidence in this developing country.

Grytdal et al. provide some of the first estimates of age-specific incidence rates for norovirus-associated acute gastroenteritis (AGE) in outpatient and community settings for the US [19]. In a population served by a managed care organization, norovirus gastroenteritis incidence in the community was estimated at 6% per year, with substantially higher rates among children under 5 years of age, particularly in outpatient settings (i.e., medically attended disease). Also for the US, Rha et al. estimated medically attended norovirus AGE rates for active-duty military personnel and their beneficiaries [20]. Like Grytdal et al., they reported that outpatient rates were approximately five times higher among children under 5 years of age compared to the rest of the population. Overall, rates of medically attended norovirus in this military population were considerably higher than estimates for the civilian population from Grytdal et al. [19] or previously published estimates [21–24], perhaps owing to more frequent exposure for military personnel.

### Molecular Epidemiology

A signature biological feature of norovirus is its genetic diversity and rapid, immune selection-driven evolution. A number of papers in this collection highlight this diversity and give insight into its mechanistic underpinnings and implications for health and disease. Most of our understanding of the molecular epidemiology of norovirus comes from outbreak samples. However, Allen et al. examined samples from sporadic cases in diverse settings: the UK and Malawi [25]. Certain GII.4 strains that caused global increases in outbreak activity could be found in sporadic samples in both of these settings many years before becoming globally predominant. Based on this, the authors suggest the importance of surveillance of sporadic disease. Such data, especially from high incidence settings in the developing world, may be crucial for understanding and anticipating strain emergence and for predicting the potential strains for vaccines.

Fumian et al. and Lee Kim et al. present data on the molecular epidemiology of norovirus outbreaks from Brazil and Australia/New Zealand, respectively [26,27]. Despite their obvious geographical differences, the observations are remarkably consistent. GII.4 viruses were identified in 72% and 63% of outbreaks in Brazil and Australia/New Zealand, respectively. Of the non-GII.4 outbreaks, the majority in both settings were inter-genotype recombinant viruses, highlighting this important evolutionary mechanism, especially for less common strains.

### Natural History, Host–Pathogen Interactions

Vomiting is a cardinal symptom of norovirus and key to its transmission, but it has also been a challenge to study directly. Using data from a collection of intentional-exposure volunteer studies, Kirby et al. show that over two-thirds of participants experience vomiting and that virus can be detected in most emesis samples from these participants [28]. Cases with vomiting but without diarrhea are typically excluded from studies of acute gastroenteritis; Kirby et al.’s study reminds us that in doing so, we exclude an important syndrome caused by norovirus infection and therefore underestimate its disease burden.

It is from these and other volunteer studies that much of our knowledge of norovirus immunity is derived. More recently, clinical trials using virus-like particles (VLPs) as candidate vaccines have further advanced our knowledge. Ramani et al. highlight two recently identified potential correlates of protection against norovirus gastroenteritis: norovirus-specific salivary IgA and norovirus-specific memory IgG cells [29]. Currently, however, serum antibody that blocks the binding of norovirus VLPs to histo-blood group antigens (HBGAs) is the best-studied and leading candidate correlate of protection.

In their Pearls article, Nordgren et al. discuss the implications of heterogeneity in innate susceptibility to norovirus in terms of burden of disease, viral genetic diversity as a function of human host diversity, and what the implications of this might be for norovirus vaccines and clinical trial design [30].

## Quantifying Disease Burden and the Need for a Vaccine

The current evidence suggests that norovirus disease burden is great, but there remains considerable uncertainty and some scientific controversy in defining the precise role of norovirus in severe pediatric gastroenteritis. Epidemiological and etiological data are lacking, especially from developing countries. Routine testing is rarely performed in ongoing surveillance platforms, in part because molecular diagnostics are the standard reference for norovirus detection, and have only recently been widely available. Real-time quantitative reverse-transcription PCR (RT-qPCR) is the most sensitive and specific diagnostic for noroviruses, but use of these assays is mainly restricted to public health and research laboratories in middle- and high-income settings.

A robust estimate of the disease burden is critical to establishing a public health case to guide interventions and norovirus vaccine development. A vexing problem is how to identify when norovirus is disease-causing and then to attribute a fraction of the acute gastroenteritis disease “envelope” to norovirus. For norovirus, attribution has been particularly challenging since detection is based on highly sensitive RT-qPCR and virus is frequently detected in stool of individuals with gastroenteritis, but also in stool of healthy controls. Reinfection is common and sometimes asymptomatic; viral shedding can persist for weeks or months after symptoms and, fundamentally, we lack a diagnostic that readily discriminates between disease-causing and asymptomatic infection.

The recent Global Enterics Multi-Center Study (GEMS) is the largest systematic assessment for understanding the etiology of childhood diarrhea in developing countries [31]. GEMS and other case-control studies use the odds ratio of a microbe being present in cases versus healthy controls to calculate an attributable or etiologic fraction. When detected as frequently in healthy controls as in cases, some study authors have concluded that norovirus is a minor pathogen. We think that the conclusion is inaccurate for a virus that commonly causes reinfection and has long excretion patterns. An alternative explanation is that high levels of asymptomatic infection are a result of frequent exposure, some of which will result in asymptomatic infection because of acquired immunity [32]. Therefore, high prevalence of norovirus detection in healthy controls may be characteristic of “hyper-endemicity” where burden is higher, not lower. Quantitative and multiplex diagnostics may be important tools for ascribing etiological fractions for norovirus and other enteric pathogens, especially when coupled with rigorous field studies, such as the multi-center MAL-ED study [33]. Indeed, in that study, norovirus was identified as the pathogen with the first- and second-highest attributable fraction for diarrhea in the first and second year of life, respectively, but still only about 5% of disease could be attributed to norovirus [6].

## Developing a Vaccine and Addressing Biological Challenges

Noroviruses are a genetically and antigenically diverse group of ssRNA viruses, which presents serious challenges both for creating broadly reactive diagnostics and eliciting a broadly protective immune response, following either natural infection or vaccination. The norovirus strains that infect humans are found among 29 genotypes among genogroup (G) I (*n* = 9), GII (*n* = 19), and GIV (*n* = 1) [34]. Among this array of different noroviruses are the GII.4 strains, which rapidly evolve in a boom-and-bust cycle, with novel viruses emerging every 2–4 years and replacing previous dominant ones, a process driven by evasion of immunity in the human population [35]. In addition to their evolutionary dynamics, there are public health reasons that a successful norovirus vaccine must provide protection against GII.4 viruses: they are the predominant cause of pediatric infections worldwide [36], they predominate overwhelmingly as a cause of disease amongst the elderly in health care–associated outbreaks, and they result in more severe illness and death [37]. Studies published in this Collection are consistent with the view that GII.4 viruses predominate globally [17,25,26], with rare exception in the last 20 years [38].

Understanding of natural immunity to norovirus is far from complete, but the current view is that immunity is strain- or genotype-specific, with little or no protection conferred across genogroups. Immunity is not lifelong, with estimates of duration ranging from 6 months to 9 years [32,39–42]. Accordingly, genotype-specific immune responses and antigenic variation suggest that a polyvalent vaccine will be needed and may require updating when new strains emerge. To date, vaccine trials and challenge studies have been conducted among adults, leaving much to learn about how “unprimed” children who have not experienced (as many) norovirus exposures develop immunity and therefore respond to vaccination.

The lack of a robust in vitro cell culture system for human norovirus has hampered the development of assays to measure protective neutralizing antibodies conferred by either natural or vaccine-induced immunity. However, important progress has been achieved very recently in this area [11] as has identification of candidate immune correlates of protection (e.g., [43]). These are important breakthroughs that may accelerate vaccine development.

## Overcoming Logistical and Programmatic Challenges

Many distinct population groups are affected by norovirus, which could complicate the formulation of a research agenda and clinical development plan for specific products (Tables 1 and 2). But, if harnessed, this diversity in disease burden could also serve to stimulate development and generate demand from difference sources. A development plan for a target population of young children will look quite different than for older adults, or for a specific risk group, such as travelers, military personnel, or health care workers. From a public health perspective, it is clear that young children experience the highest overall incidence of disease [21], and severe disease outcomes are most common among young children and, at least in developed countries, the elderly [24]. Young children also seem to be the most important group in driving transmission in the community [44]. Accordingly, vaccinating young children would likely be most efficient for directly preventing disease burden, and would offer the greatest potential for impact at the population level through indirect benefits resulting from reduced transmission. Defining the relative roles of different age groups in transmission is challenging and may well differ in high- and low-income settings, but a combination of empirical (e.g., household) studies and mathematical modeling analysis may help to optimize the direct and population-level effect of vaccinating through targeting different groups.

**Table 1:** Epidemiological and economics characteristics of various age groups for considering norovirus vaccines^1^ ^,^ ^2^.

	Incidence	Health care utilization^3^	Hospitalization	Deaths^4^	Societal costs	Health care costs	Role/risk in transmission	Challenges in vaccinating: immunological	Challenges in vaccinating: programmatic
***Children (<5 years)***	High	High	High	Med.	High	High	High	Naïve: may need multiple doses	Interaction with other routine immunizations
***Older children (5–14 years)***	Med.	Low	Low	Low	Med.	Low	Med.	History of exposure	
***Younger adults (15–64 years)***	Med.	Low	Low	Low	Med.	Low	Med.	History of exposure	Generally low coverage
***Older adults (≥65 years)***	Low	Med.	High	High	Low	High	Low	History of exposure immune senescence	Generally low coverage

^1^ For all groups, revaccination may be required after strain shifts.

^2^ Rankings (low/medium/high) are subjective and relative to norovirus disease only—no comparisons to other diseases are intended.

^3^ Outpatient, emergency, and ambulatory services

^4^ Little data from lower income settings.

**Table 2:** Epidemiological, economic, and programmatic considerations for specific subpopulation risk groups.

	Health care workers	Travelers	Military personnel	Immunocompromised	Food service workers
***Incidence***	Affected in outbreaks	High	High	Unknown	Likely same as general population
***Severe disease***	Low risk	Can have limited access to care while traveling	Outbreaks may occur under extreme temperature and exertional conditions	High risk, including chronic infection and death	Low risk
***Economic losses and disruption***	Productivity losses; compromised patient care from missed work	Loss of personal travel funds, sometimes loss to operators (e.g., cruise industry)	Impact on training, mission readiness, and operations	Costly extended length of hospitalization	Productivity losses from missed work; impact on business of product recall, store closure, or brand impact
***Role in transmission and potential indirect benefits of vaccination***	May transmit to patients, but current evidence suggests low rates	Generally low, but potential for transmitting on aircraft, buses, hotels, etc.	Potentially high for those resident in barracks, on ships, or on missions	Unknown, but potential risk due to prolonged shedding	High: food handlers implicated in the majority of foodborne norovirus outbreaks
***Challenges in vaccinating*: *immunological***	May have extensive history of exposure	Unfamiliar strains during foreign travel	Unfamiliar strains during foreign deployment	Poor immune response	None
***Challenges in vaccinating*: *programmatic***	Has taken many years to achieve reasonable influenza vaccine coverage in the United States; Many health care workers do not get vaccinated.	Need to be vaccinated in a travel clinic, with sufficient time to mount immune response before departure	None, but if given in recruit setting, interference with other concomitant immunizations should be assessed	May be difficult to identify in advance of exposure	Hard to reach population with high turnover; unwillingness of employer to pay

In the absence of an outside stimulus, such as major global public health donors, developed world markets are likely to provide the initial economic impetus for private industry to develop norovirus vaccines. To date, early-phase trials have been conducted among adults in high-income settings, and there have been no clinical studies in children, so we see a need for a targeted pediatric development plan. Such a plan should include studies to generate data on the compatibility of a norovirus vaccine with current routine childhood immunizations, and in particular the Expanded Program on Immunization (EPI). Adding a vaccine to the EPI schedule involves great effort to demonstrate the added value of the vaccine, on both economic and health grounds. The economics of a norovirus vaccine requiring multiple doses and/or periodic reformulation will be scrutinized carefully by policy makers. At the earliest stage, clinical development plans should define a target product profile (TPP) that will maximize public health gains by focusing on young children, with the aim of developing a vaccine that can be incorporated into the logistical arrangements of current immunization programs.

## Future Directions

Norovirus as a target for vaccination is unique in many ways. First, the public health need is not restricted to a specific region, income level, or even age range, so the groups with a stake in vaccine development are many and diverse. Second, much of the totality of economic and health burden results from relatively mild disease. But even if the proportion of cases with severe outcomes is relatively low, the sheer incidence of norovirus still results in a considerable severe disease burden. Third, given the current state of knowledge, we expect that progress in understanding of disease burden and epidemiology, human–virus interactions, and vaccine development will be interdependent. More robust estimates of the disease burden of children in low-income settings should stimulate more research in vaccines that will benefit those populations; vaccine trials themselves can be used to better characterize the disease burden and also to identify correlates of protection, which have the potential to make subsequent vaccine evaluations faster and less costly; mathematical modeling studies can serve as a framework to integrate this variety of data and to predict the impact of vaccination strategies, and to objectively identify the most critical gaps in our knowledge (Table 3). Addressing these key issues will be vital to accelerate and achieve the development and implementation of interventions such as vaccines to control and prevent the tremendous global morbidity and mortality from norovirus.

**Table 3:** Critical studies to be performed and questions to be answered to advance vaccine development.

	Epidemiology and disease burden	Diagnostics and strain surveillance	Immunity and susceptibility	Vaccine development	Logistical and programmatic
Studies optimally designed for norovirus (in terms of diagnostics and case definitions) to more definitively quantify the incidence and burden, including severe disease, especially in lower-income settings.	●	●			
Development and optimization of diagnostics for use in etiological studies and clinical trials.	●	●		●	
Birth cohort studies in low-, middle-, and high-income settings to further understanding of the acquisition of immunity.	●		●		
Development of a Global Norovirus Surveillance Network to characterize worldwide strain distribution and evolutionary dynamics.		●			
Evaluations and reproducibility of in vitro cell culture system candidates.			●	●	
Confirmation of currently proposed immune correlates of protection and their validation in different populations.			●	●	
Human clinical studies to characterize the safety, immunogenicity, and efficacy of products not yet trialed in humans.				●	
Pivotal, phase III field efficacy studies to demonstrate protection against disease in the community.				●	
A probe study, once a vaccine is available, to simultaneously define the vaccine performance and disease burden.	●			●	●
Mathematical modeling studies to examine the direct and population-level effect of vaccinating different groups, defined by age or risk profile, including economic evaluation for settings in developing countries.	●				●
Development of a target product profile for a vaccine to be used in the EPI schedule.				●	●

## Abbreviations

AGE: acute gastroenteritis
BMGF: the Bill & Melinda Gates Foundation
CDC: Centers for Disease Control and Prevention
DALYs: disability-adjusted life years
EPI: Expanded Program on Immunization
G: genogroup
GEMS: Global Enterics Multi-Center Study
HBGAs: histo-blood group antigens
MAL-ED: Malnutrition and Enteric Disease Study
RT-qPCR: real-time quantitative PCR
TPP: target product profile
VLPs: virus like particles

## References

[1] GBD 2013 Mrtality and Causes of Death Collaborators. Global, regional, and national age-sex specific all-cause and cause-specific mortality for 240 causes of death, 1990–2013: a systematic analysis for the Global Burden of Disease Study 2013. Lancet. 2015;385(9963):117–71. 10.1016/S0140-6736(14)61682-2 25530442 PMC4340604

[2] Liu L, Oza S, Hogan D, Perin J, Rudan I, Lawn JE, et al Global, regional, and national causes of child mortality in 2000–13, with projections to inform post-2015 priorities: an updated systematic analysis. Lancet. 2015;385(9966):430–40. 10.1016/S0140-6736(14)61698-6 . 25280870

[3] Global Health Data Exchange: Institute for Health Metrics and Evaluation; 2013. http://vizhub.healthdata.org/irank/arrow.php.

[4] Yen C, Tate JE, Hyde TB, Cortese MM, Lopman BA, Jiang B, et al Rotavirus vaccines: current status and future considerations. Human vaccines & immunotherapeutics. 2014;10(6):1436–48. 10.4161/hv.28857 . 24755452 PMC4185955

[5] Pires SM, Fischer-Walker CL, Lanata CF, Devleesschauwer B, Hall AJ, Kirk MD, et al Aetiology-Specific Estimates of the Global and Regional Incidence and Mortality of Diarrhoeal Diseases Commonly Transmitted through Food. PLoS ONE. 2015;10(12):e0142927 10.1371/journal.pone.0142927 26632843 PMC4668836

[6] Platts-Mills JA, Babji S, Bodhidatta L, Gratz J, Haque R, Havt A, et al Pathogen-specific burdens of community diarrhoea in developing countries: a multisite birth cohort study (MAL-ED). The Lancet Global health. 2015;3(9):e564–75. 10.1016/S2214-109X(15)00151-5 . 26202075 PMC7328884

[7] Bucardo F, Reyes Y, Svensson L, Nordgren J. Predominance of norovirus and sapovirus in Nicaragua after implementation of universal rotavirus vaccination. PLoS ONE. 2014;9(5):e98201 10.1371/journal.pone.0098201 24849288 PMC4029982

[8] Payne DC, Vinje J, Szilagyi PG, Edwards KM, Staat MA, Weinberg GA, et al Norovirus and medically attended gastroenteritis in U.S. children. N Engl J Med. 2013;368(12):1121–30. 10.1056/NEJMsa1206589 23514289 PMC4618551

[9] Ahmed SM, Hall AJ, Robinson AE, Verhoef L, Premkumar P, Parashar UD, et al Global prevalence of norovirus in cases of gastroenteritis: a systematic review and meta-analysis. Lancet Infect Dis. 2014;14(8):725–30. 10.1016/S1473-3099(14)70767-4 . 24981041 PMC8006533

[10] Kirk MD, Pires SM, Black RE, Caipo M, Crump JA, Devleesschauwer B, et al World Health Organization Estimates of the Global and Regional Disease Burden of 22 Foodborne Bacterial, Protozoal, and Viral Diseases, 2010: A Data Synthesis. PLoS Med. 2015;12(12):e1001921 10.1371/journal.pmed.1001921 26633831 PMC4668831

[11] Jones MK, Watanabe M, Zhu S, Graves CL, Keyes LR, Grau KR, et al Enteric bacteria promote human and mouse norovirus infection of B cells. Science. 2014;346(6210):755–9. 10.1126/science.1257147 25378626 PMC4401463

[12] Baldridge MT, Nice TJ, McCune BT, Yokoyama CC, Kambal A, Wheadon M, et al Commensal microbes and interferon-lambda determine persistence of enteric murine norovirus infection. Science. 2015;347(6219):266–9. 10.1126/science.1258025 25431490 PMC4409937

[13] Osborne LC, Monticelli LA, Nice TJ, Sutherland TE, Siracusa MC, Hepworth MR, et al Coinfection. Virus-helminth coinfection reveals a microbiota-independent mechanism of immunomodulation. Science. 2014;345(6196):578–82. 10.1126/science.1256942 25082704 PMC4548887

[14] Atmar RL, Bernstein DI, Harro CD, Al-Ibrahim MS, Chen WH, Ferreira J, et al Norovirus vaccine against experimental human Norwalk Virus illness. N Engl J Med. 2011;365(23):2178–87. Epub 2011/12/14. 10.1056/NEJMoa1101245 . 22150036 PMC3761795

[15] Bernstein DI, Atmar RL, Lyon GM, Treanor JJ, Chen WH, Jiang X, et al Norovirus vaccine against experimental human GII.4 virus illness: a challenge study in healthy adults. J Infect Dis. 2015;211(6):870–8. 10.1093/infdis/jiu497 . 25210140 PMC5914500

[16] Bartsch SM, Lopman BA, Ozawa S, Hall AJ, Lee BY. Global Economic Burden of Norovirus Gastroenteritis. PLoS ONE. 2016;11(4):e0151219 10.1371/journal.pone.0151219 27115736 PMC4846012

[17] Mans J, Armah GE, Steele AD, Taylor MB. Norovirus Epidemiology in Africa: A Review. PLoS ONE. 2016;11(4):e0146280 10.1371/journal.pone.0146280 27116615 PMC4846019

[18] Shioda K, Cosmas L, Audi A, Gregoricus N, Vinjé J, Parashar UD, et al Population-Based Incidence Rates of Diarrheal Disease Associated with Norovirus, Sapovirus, and Astrovirus in Kenya. PLoS ONE. 2016;11(4):e0145943 10.1371/journal.pone.0145943 27116458 PMC4845984

[19] Grytdal SP, DeBess E, Lee LE, Blythe D, Ryan P, Biggs C, et al Incidence of Norovirus and Other Viral Pathogens That Cause Acute Gastroenteritis (AGE) among Kaiser Permanente Member Populations in the United States, 2012–2013. PLoS ONE. 2016;11(4):e0148395 10.1371/journal.pone.0148395 27115485 PMC4846013

[20] Rha B, Lopman BA, Alcala AN, Riddle MS, Porter CK. Incidence of Norovirus-Associated Medical Encounters among Active Duty United States Military Personnel and Their Dependents. PLoS ONE. 2016;11(4):e0148505 10.1371/journal.pone.0148505 27115602 PMC4845987

[21] Phillips G, Tam CC, Conti S, Rodrigues LC, Brown D, Iturriza-Gomara M, et al Community incidence of norovirus-associated infectious intestinal disease in England: improved estimates using viral load for norovirus diagnosis. Am J Epidemiol. 2010;171(9):1014–22. Epub 2010/04/03. 10.1093/aje/kwq021 . 20360244

[22] Verhoef L, Koopmans M, VANP W, Duizer E, Haagsma J, Werber D, et al The estimated disease burden of norovirus in The Netherlands. Epidemiol Infect. 2013;141(3):496–506. Epub 2012/05/19. 10.1017/S0950268812000799 . 22595489 PMC9151884

[23] Tam CC, Rodrigues LC, Viviani L, Dodds JP, Evans MR, Hunter PR, et al Longitudinal study of infectious intestinal disease in the UK (IID2 study): incidence in the community and presenting to general practice. Gut. 2012;61(1):69–77. 10.1136/gut.2011.238386 21708822 PMC3230829

[24] Hall AJ, Lopman BA, Payne DC, Patel MM, Gastanaduy PA, Vinje J, et al Norovirus disease in the United States. Emerg Infect Dis. 2013;19(8):1198–205. 10.3201/eid1908.130465 23876403 PMC3739528

[25] Allen DJ, Trainor E, Callaghan A, O'Brien SJ, Cunliffe NA, Iturriza-Gómara M. Early Detection of Epidemic GII-4 Norovirus Strains in UK and Malawi: Role of Surveillance of Sporadic Acute Gastroenteritis in Anticipating Global Epidemics. PLoS ONE. 2016;11(4):e0146972 10.1371/journal.pone.0146972 27115152 PMC4846118

[26] Fumian TM, da Silva Ribeiro de Andrade J, Leite JPG, Miagostovich MP. Norovirus Recombinant Strains Isolated from Gastroenteritis Outbreaks in Southern Brazil, 2004–2011. PLoS ONE. 2016;11(4):e0145391 10.1371/journal.pone.0145391 27116353 PMC4846083

[27] Lim KL, Hewitt J, Sitabkhan A, Eden J-S, Lun J, Levy A, et al A Multi-Site Study of Norovirus Molecular Epidemiology in Australia and New Zealand, 2013–2014. PLoS ONE. 2016;11(4):e0145254 10.1371/journal.pone.0145254 27116221 PMC4846056

[28] Kirby AE, Streby A, Moe CL. Vomiting as a Symptom and Transmission Risk in Norovirus Illness: Evidence from Human Challenge Studies. PLoS ONE. 2016;11(4):e0143759 10.1371/journal.pone.0143759 27116105 PMC4845978

[29] Ramani S, Estes MK, Atmar RL. Correlates of Protection against Norovirus Infection and Disease—Where Are We Now, Where Do We Go? PLoS Pathog. 12(4): e1005334 10.1371/journal.ppat.1005334 PMC4846004 27115358

[30] Nordgren J, Sharma S, Kambhampati A, Lopman BA, Svensson L. Innate Resistance and Susceptibility to Norovirus Infection. PLoS Pathog. 12(4): e1005385 10.1371/journal.ppat.1005385 PMC4845991 27115484

[31] Kotloff KL, Nataro JP, Blackwelder WC, Nasrin D, Farag TH, Panchalingam S, et al Burden and aetiology of diarrhoeal disease in infants and young children in developing countries (the Global Enteric Multicenter Study, GEMS): a prospective, case-control study. Lancet. 2013;382(9888):209–22. 10.1016/S0140-6736(13)60844-2 . 23680352

[32] Lopman B, Simmons K, Gambhir M, Vinje J, Parashar U. Epidemiologic implications of asymptomatic reinfection: a mathematical modeling study of norovirus. Am J Epidemiol. 2014;179(4):507–12. 10.1093/aje/kwt287 . 24305574

[33] Platts-Mills JA, McCormick BJ, Kosek M, Pan WK, Checkley W, Houpt ER, et al Methods of analysis of enteropathogen infection in the MAL-ED Cohort Study. Clin Infect Dis. 2014;59 Suppl 4:S233–8. 10.1093/cid/ciu408 25305292 PMC4204610

[34] Vinje J. Advances in laboratory methods for detection and typing of norovirus. J Clin Microbiol. 2015;53(2):373–81. 10.1128/JCM.01535-14 24989606 PMC4298492

[35] Siebenga JJ, Vennema H, Renckens B, de Bruin E, van der Veer B, Siezen RJ, et al Epochal evolution of GGII.4 norovirus capsid proteins from 1995 to 2006. J Virol. 2007;81(18):9932–41. 10.1128/JVI.00674-07 17609280 PMC2045401

[36] Hoa Tran TN, Trainor E, Nakagomi T, Cunliffe NA, Nakagomi O. Molecular epidemiology of noroviruses associated with acute sporadic gastroenteritis in children: global distribution of genogroups, genotypes and GII.4 variants. J Clin Virol. 2013;56(3):185–93. 10.1016/j.jcv.2012.11.011 . 23218993

[37] Desai R, Hembree CD, Handel A, Matthews JE, Dickey BW, McDonald S, et al Severe outcomes are associated with genogroup 2 genotype 4 norovirus outbreaks: a systematic literature review. Clin Infect Dis. 2012;55(2):189–93. 10.1093/cid/cis372 22491335 PMC3491774

[38] de Graaf M, van Beek J, Vennema H, Podkolzin AT, Hewitt J, Bucardo F, et al Emergence of a novel GII.17 norovirus—End of the GII.4 era? Euro Surveill. 2015;20(26). . 26159308 10.2807/1560-7917.es2015.20.26.21178 PMC5921880

[39] Baron RC, Greenberg HB, Cukor G, Blacklow NR. Serological responses among teenagers after natural exposure to Norwalk virus. JInfectDis. 1984;150(4):531–4. 10.1093/infdis/150.4.531 6092484

[40] Johnson PC, Mathewson JJ, DuPont HL, Greenberg HB. Multiple-challenge study of host susceptibility to Norwalk gastroenteritis in US adults. J Infect Dis. 1990;161(1):18–21. . 2153184 10.1093/infdis/161.1.18

[41] Parrino TA, Schreiber DS, Trier JS, Kapikian AZ, Blacklow NR. Clinical immunity in acute gastroenteritis caused by Norwalk agent. N Engl J Med. 1977;297(2):86–9. 10.1056/NEJM197707142970204 . 405590

[42] Wyatt RG, Dolin R, Blacklow NR, DuPont HL, Buscho RF, Thornhill TS, et al Comparison of three agents of acute infectious nonbacterial gastroenteritis by cross-challenge in volunteers. J Infect Dis. 1974;129(6):709–14. Epub 1974/06/01. . 4209723 10.1093/infdis/129.6.709

[43] Atmar RL, Bernstein DI, Lyon GM, Treanor JJ, Al-Ibrahim MS, Graham DY, et al Serological Correlates of Protection against a GII.4 Norovirus. Clin Vaccine Immunol. 2015;22(8):923–9. 10.1128/CVI.00196-15 26041041 PMC4519714

[44] de Wit MA, Koopmans MP, van Duynhoven YT. Risk factors for norovirus, Sapporo-like virus, and group A rotavirus gastroenteritis. Emerg Infect Dis. 2003;9(12):1563–70. 10.3201/eid0912.020076 14720397 PMC3034344

